# Hypoxia‐Associated Molecular Subtypes Reveal Immune Checkpoint, Ferroptosis, and m6A Regulatory Heterogeneity in Pediatric Vasculitis

**DOI:** 10.1155/humu/7451086

**Published:** 2026-06-26

**Authors:** Bin Liu

**Affiliations:** ^1^ Department of Vascular Surgery, Affiliated Hospital of Jiangsu University, Zhenjiang, China, ujs.edu.cn; ^2^ Department of Vascular Surgery, Affiliated Hospital, Southwest Medical University, Luzhou, China, swmu.edu.cn

**Keywords:** ferroptosis, germline mutation, hypoxia, immune checkpoints, m6A methylation, pediatric vasculitis, vasculitis

## Abstract

**Background:**

Vasculitis is a heterogeneous inflammatory vascular disorder with substantial clinical and molecular diversity. However, hypoxia‐associated molecular subtypes in pediatric vasculitis and their differences from adult vasculitis remain poorly defined.

**Methods:**

Transcriptomic data of pediatric and adult vasculitis were obtained from the GEO database. RNA‐seq data from GSE129752 were normalized by log_2_ (TPM+1) transformation. Hypoxia‐related genes were used for consensus clustering of pediatric vasculitis samples. Immune checkpoint, ferroptosis‐related, and m6A methylation regulator genes were compared between subtypes. Differentially expressed genes between pediatric and adult vasculitis were identified using limma, followed by Gene Ontology enrichment analysis using clusterProfiler.

**Results:**

Consensus clustering separated pediatric vasculitis into two hypoxia‐associated subtypes, high hypoxia pediatric vasculitis population (high_hypoxia group) and low hypoxia pediatric vasculitis population (low_hypoxia group), with distinct transcriptomic profiles. High_hypoxia group showed higher expression of multiple immune checkpoint genes, including HAVCR2, IGSF8, ITPRIPL1, LAG3, PDCD1, SIGLEC15, and TIGIT. Ferroptosis‐related genes EMC2 and ATP5MC3 were also elevated in the high_hypoxia group. In addition, most m6A regulators, including FTO, METTL14, WTAP, and RBM15, were significantly upregulated in the high_hypoxia group. Comparison between pediatric and adult vasculitis identified 85 differentially expressed genes, including 21 upregulated and 64 downregulated genes in pediatric vasculitis. Functional enrichment suggested that pediatric vasculitis was associated with fibroblast proliferation and vascular remodeling, whereas adult vasculitis was enriched in immature T‐cell regulation and fatty acid transport. Germline mutation–related genes, including VHL, BRCA1, RET, and MUTYH, showed coordinated correlations with age‐related vasculitis.

**Conclusion:**

Hypoxia‐associated molecular heterogeneity exists in pediatric vasculitis and we observed age‐related transcriptomic differences between pediatric and adult disease. These findings provide a foundation for precision classification and future mechanism‐based therapeutic strategies.

## 1. Introduction

Vasculitis comprises a heterogeneous group of inflammatory vascular disorders characterized by immune‐mediated injury of blood vessel walls, leading to endothelial dysfunction, luminal narrowing, thrombosis, aneurysm formation, and tissue ischemia [1, 2]. Depending on the size and distribution of affected vessels, vasculitis is commonly classified into large‐, medium‐, small‐, and variable‐vessel vasculitis, with diverse clinical manifestations ranging from mild cutaneous lesions to severe multisystem organ damage [3–5]. Current clinical management mainly relies on glucocorticoids, conventional immunosuppressants, biologic agents, and targeted anti‐inflammatory therapies [6, 7]. Although these strategies have improved disease control, many patients still experience relapse, treatment‐related toxicity, incomplete response, or long‐term vascular complications [8, 9]. Therefore, identifying molecular subtypes and disease‐driving mechanisms is essential for improving risk stratification and precision therapy [10, 11].

Previous studies have shown that vasculitis progression is driven by complex interactions among endothelial injury, innate and adaptive immune activation, cytokine release, complement activation, neutrophil extracellular trap formation, oxidative stress, and vascular remodeling [12, 13]. Classical inflammatory pathways, including NF‐*κ*B, JAK/STAT, MAPK, PI3K–AKT, TLR signaling, and inflammasome activation, regulate leukocyte recruitment, endothelial activation, cytokine amplification, and tissue damage [14, 15]. Hypoxia is another critical feature of inflamed vascular tissues, where impaired perfusion and inflammatory cell infiltration can reshape endothelial metabolism, immune responses, and vascular remodeling [16–18]. Hypoxia‐associated molecular typing provides a useful framework for classifying vasculitis according to oxygen‐stress–responsive transcriptional programs rather than clinical phenotype alone [19]. In inflamed vessels, endothelial swelling, immune‐cell infiltration, microthrombosis, and impaired perfusion create a hypoxic microenvironment that stabilizes HIF‐1*α* and HIF‐2*α* [20, 21]. These factors promote glycolytic reprogramming, VEGF‐mediated vascular responses, endothelial adhesion molecule expression, and inflammatory cytokine production, including IL‐1*β*, IL‐6, TNF‐*α*, CXCL8, CCL2, and CXCL10 [22–24]. Hypoxia interacts with NF‐*κ*B, MAPK, and TLR signaling, thereby reinforcing endothelial activation, neutrophil recruitment, macrophage polarization, T cell dysfunction, and vascular remodeling [25, 26].

In addition, hypoxia also coordinates immune checkpoint activation, ferroptosis, and N6‐methyladenosine (m6A) RNA methylation [27]. Persistent antigenic stimulation, endothelial injury, inflammatory cytokines, and HIF‐dependent transcriptional programs can induce inhibitory checkpoint molecules such as PDCD1/PD‐1, TIM‐3, and LAG3 in T cells, myeloid cells, and endothelial‐interacting immune populations [28–30]. These molecules may limit excessive immune activation but can also indicate immune exhaustion, impaired inflammatory resolution, and persistent tissue injury [31–33]. Ferroptosis provides another mechanistic link between hypoxia and vascular damage. Ferroptosis is an iron‐dependent form of regulated cell death driven by excessive lipid peroxidation and impaired antioxidant defense [34–36]. Iron overload, mitochondrial stress, reactive oxygen species accumulation, and dysfunction of antioxidant pathways such as SLC7A11–GSH–GPX4 can trigger lipid peroxidation‐dependent endothelial injury and sterile inflammation [37, 38]. In parallel, m6A regulators modulate mRNA stability and translation of genes involved in HIF signaling, cytokine responses, immune‐cell differentiation, and ferroptosis sensitivity [39, 40]. However, whether hypoxia‐related molecular states interact with immune checkpoint activity, ferroptosis, and m6A regulation in vasculitis, especially pediatric vasculitis, remains insufficiently understood [41, 42].

Pediatric vasculitis differs from adult vasculitis in disease spectrum, immune maturity, clinical presentation, organ involvement, treatment response, and long‐term prognosis [43–45]. Currently, most mechanistic studies have focused on adult populations, and relatively few investigations have systematically compared pediatric and adult vasculitis at the transcriptomic level [46, 47]. Moreover, whether pediatric vasculitis contains distinct hypoxia‐related molecular subtypes remains largely unexplored. In this study, we integrated transcriptomic data to identify hypoxia‐associated subgroups in pediatric vasculitis and further characterized their immune checkpoint, ferroptosis, and m6A methylation profiles. We also compared pediatric and adult vasculitis to define age‐related gene expression signatures and enriched biological pathways. This work provides a molecular framework for understanding vasculitis heterogeneity and may support future precision classification and therapeutic development.

## 2. Methods

### 2.1. Data Acquisition and Preprocessing

All transcriptomic datasets used in this study were obtained from the Gene Expression Omnibus (GEO) database (https://www.ncbi.nlm.nih.gov/geo/). Quantile normalization was conducted using the normalize.quantiles function from the preprocessCore R package. For high‐throughput sequencing data, normalized RNA‐seq counts from GSE129752 were downloaded in TPM format and further transformed using log_2_(TPM+1) prior to downstream analyses. After preprocessing and quality control, transcriptomic data from 32 pediatric vasculitis samples and 11 adult vasculitis samples were included for subsequent comparative and molecular subtype analyses.

### 2.2. Consensus Clustering for Molecular Subtype Identification

To identify hypoxia‐associated molecular subtypes in pediatric vasculitis, consensus clustering was performed using the ConsensusClusterPlus R package. Consensus clustering was selected because it evaluates the robustness and reproducibility of molecular subtypes through repeated subsampling, clustering, and consensus matrix construction, thereby reducing the instability that may arise from a single unsupervised clustering procedure. The maximum number of clusters was set to 6 because the pediatric cohort size was limited, and a larger number of clusters could lead to biologically uninterpretable or statistically unstable subgroups. For each clustering iteration, 80% of samples were randomly resampled, and 100 resampling iterations were performed to assess clustering stability. Hierarchical clustering with Ward.D2 linkage was used to minimize within‐cluster variance and is commonly applied for transcriptomic subtype discovery. The optimal number of clusters was selected by jointly considering the consensus cumulative distribution function (CDF) curve, the relative change in the area under the CDF curve, the consensus matrix heatmap, and the proportion of ambiguous clustering score. A lower proportion of ambiguous clustering indicates greater subtype stability. Based on these criteria, *k* = 2 was selected as the optimal clustering solution because it showed stable within‐cluster consistency, clear between‐cluster separation, and biologically interpretable hypoxia‐associated transcriptional differences.

### 2.3. Heatmap Visualization and Expression Profiling

Hierarchical clustering heatmaps were generated using the pheatmap R package. For genome‐wide heatmap visualization, genes with variance ≤ 0.1 across samples were excluded because genes with minimal expression variation provide limited information for distinguishing sample‐level transcriptional patterns and may increase visual noise. This variance threshold was applied only for dimensionality reduction and visualization, not for statistical inference. When the number of retained genes exceeded 1000, genes were ranked by variance, and the Top 25% most variable genes were selected for heatmap display. This strategy allowed clearer visualization of dominant transcriptional heterogeneity while avoiding overinterpretation of low‐variance genes.

### 2.4. Functional Gene Set Analysis

To characterize biological differences between molecular subtypes, curated gene sets associated with immune checkpoints, ferroptosis, and m6A RNA methylation regulators were collected from published literature and public databases. Differential expression patterns of these functional gene sets were compared between identified pediatric vasculitis subtypes.

### 2.5. Differential Expression Analysis

Differentially expressed genes (DEGs) between pediatric and adult vasculitis cohorts were identified using the limma R package (Version 3.40.2). The limma method was selected because it uses linear modeling combined with empirical Bayes moderation, which improves variance estimation and statistical reliability in high‐dimensional transcriptomic analyses, particularly when sample sizes are relatively limited. Adjusted *p* value was calculated to control the false discovery rate using the Benjamini–Hochberg method. Genes meeting the criteria of adjusted *p* < 0.05 and |log_2_ fold change| > 1 were considered statistically significant DEGs. Volcano plots were used to visualize differential expression results, where the *x*‐axis represented log_2_ fold change and the *y*‐axis represented –log10(adjusted *p* value).

### 2.6. Functional Enrichment Analysis

Gene Ontology (GO) enrichment analysis was performed using the clusterProfiler R package. This method provides a standardized statistical framework for identifying overrepresented biological processes, molecular functions, and cellular components among DEGs. Enrichment results were visualized using bar plots, bubble plots, and circular plots to facilitate biological interpretation.

### 2.7. Statistical Analysis

All statistical analyses were conducted using R software Version 4.0.3. For comparisons between two groups, continuous variables were assessed using parametric or nonparametric tests according to data distribution and variance homogeneity. Normally distributed data were compared using Student′s *t*‐test, whereas nonnormally distributed data were compared using the Wilcoxon rank‐sum test. For transcriptome‐wide analyses, multiple‐testing correction was performed using the Benjamini–Hochberg method to control the false discovery rate. *p* < 0.05 was considered statistically significant.

## 3. Results

### 3.1. Identification of Hypoxia‐Associated Molecular Subtypes in Pediatric Vasculitis

**Figure 1:**
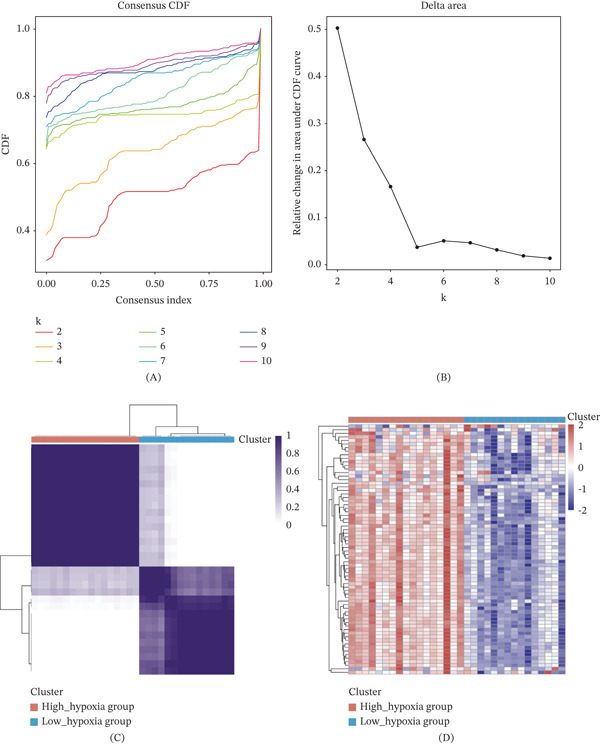
Identification of hypoxia‐related molecular subtypes in pediatric vasculitis by consensus clustering. (A) Consensus cumulative distribution function (CDF) plots for cluster numbers ranging from *k* = 2 to *k* = 10. (B) Relative change in the area under the CDF curve (delta area plot) used to determine the optimal number of clusters. (C) Consensus matrix heatmap for *k* = 2, showing clear separation of samples into two stable clusters (high_hypoxia group and low_hypoxia group). Darker colors indicate higher consensus values. (D) Unsupervised hierarchical clustering heatmap of representative genes distinguishing the two pediatric vasculitis subtypes. Columns represent patient samples and rows represent genes. Red indicates relatively high expression, whereas blue indicates relatively low expression.

Recent advances in precision medicine have emphasized the importance of molecular stratification within clinically heterogeneous diseases. Given that hypoxic stress profoundly influences vascular homeostasis, we sought to classify pediatric vasculitis patients based on hypoxia‐related transcriptional signatures. Using the GSE129752 RNA‐seq cohort, consensus clustering was performed with the ConsensusClusterPlus package. The CDF curves and relative change in area under the CDF suggested that **k** = 2 represented the optimal clustering solution (Figure 1A,B). Accordingly, pediatric vasculitis patients were robustly divided into two molecular subtypes, designated high_hypoxia group and low_hypoxia group.

Consensus matrix analysis demonstrated high intracluster similarity and low intercluster overlap, indicating stable classification performance (Figure 1C). Furthermore, unsupervised hierarchical clustering heatmap analysis revealed substantial transcriptomic divergence between the two subgroups, confirming marked heterogeneity in global gene expression profiles (Figure 1D). These findings indicate that hypoxia‐associated molecular patterns can effectively stratify pediatric vasculitis into biologically distinct subtypes.

### 3.2. Distinct Immune Checkpoint and Ferroptosis Landscapes Between Hypoxia‐Associated Pediatric Vasculitis Subtypes

**Figure 2:**
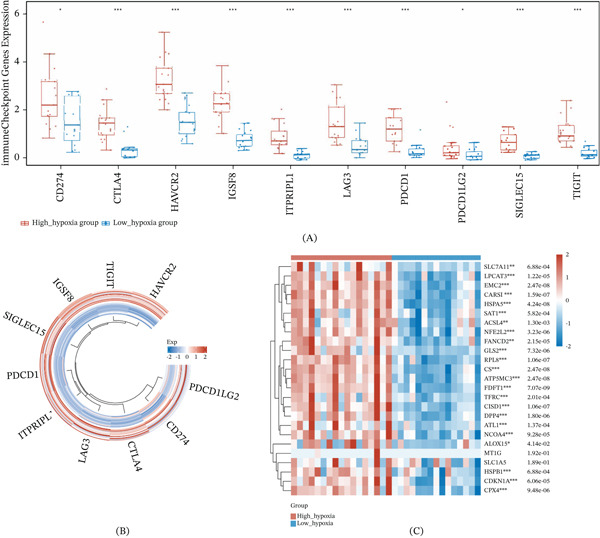
Differential immune checkpoint and ferroptosis signatures between hypoxia‐associated pediatric vasculitis subtypes. (A) Comparative expression analysis of immune checkpoint‐related genes between pediatric vasculitis subtypes. (B) Circular heatmap illustrating the global expression patterns of immune checkpoint genes across individual samples in the high_hypoxia group and low_hypoxia group. Red indicates relatively high expression and blue indicates relatively low expression. (C) Heatmap of ferroptosis‐related genes differentially expressed between pediatric vasculitis subtypes.

Aberrant immune checkpoint signaling has been increasingly implicated in the pathological progression of vasculitis. To further characterize the immunological differences between the two hypoxia‐associated pediatric vasculitis subtypes, we compared the expression profiles of representative immune checkpoint genes between the high_hypoxia group and low_hypoxia group. Boxplot analysis demonstrated that multiple inhibitory checkpoint molecules, including HAVCR2, IGSF8, ITPRIPL1, LAG3, PDCD1, SIGLEC15, and TIGIT, were significantly upregulated in the high_hypoxia group subgroup compared with the low_hypoxia group (Figure 2A). These findings were further visualized by circular heatmap analysis, which highlighted a coordinated elevation of immune checkpoint signatures in the high_hypoxia group (Figure 2B). Collectively, these results suggest that the high_hypoxia group may exhibit a more immunologically suppressed or exhausted immune microenvironment than the low_hypoxia group. Programmed cell death is a critical component of vascular inflammation, and ferroptosis has recently emerged as an important contributor to vasculitic tissue injury. Therefore, we next investigated ferroptosis‐related gene expression patterns between the two subtypes. Heatmap analysis revealed that EMC2 and ATP5MC3 were significantly elevated in the high_hypoxia group relative to the low_hypoxia group, whereas several additional ferroptosis regulators displayed subtype‐dependent trends (Figure 2C). These data indicate that hypoxia‐associated pediatric vasculitis subtypes may differ not only in immune checkpoint activity but also in ferroptosis‐related metabolic vulnerability.

### 3.3. Differential Expression of m6A‐Related Regulators Between Hypoxia‐Associated Pediatric Vasculitis Subtypes

**Figure 3:**
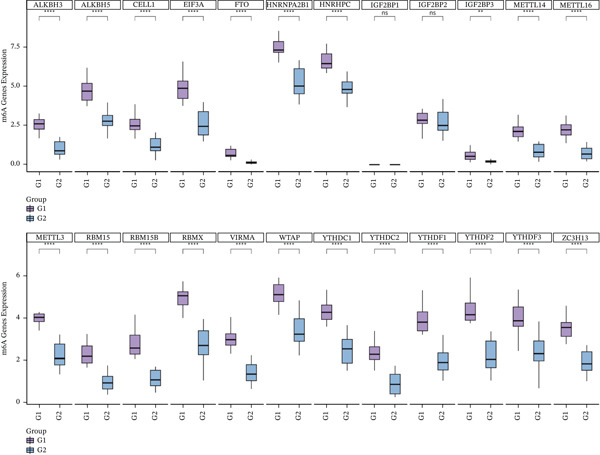
Differential expression of m6A‐related regulators between high_hypoxia and low_hypoxia pediatric vasculitis subtypes. G1 means “high_hypoxia group”, G2 means “low_hypoxia group”.

Given the emerging role of m6A RNA modification in inflammatory vascular diseases, we further examined whether m6A‐related regulators differed between high_hypoxia and low_hypoxia pediatric vasculitis groups. Comparative expression analysis showed that most m6A regulators were significantly upregulated in the high_hypoxia pediatric vasculitis subgroup compared with the low_hypoxia group. In particular, FTO, METTL14, WTAP, RBM15, RBM15B, YTHDC1, YTHDF1, YTHDF2, and ZC3H13 displayed markedly higher expression in the high_hypoxia group (Figure 3). Notably, several core m6A writers, erasers, and readers were simultaneously elevated in the high_hypoxia group, suggesting that this subtype may possess a more active RNA methylation regulatory state. In contrast, IGF2BP1 and IGF2BP2 showed no significant difference between the two groups. These findings indicate that hypoxia‐associated pediatric vasculitis subtypes may differ substantially in posttranscriptional regulatory programs, particularly m6A‐mediated RNA modification.

### 3.4. Identification of Distinct Transcriptomic Signatures Between Pediatric and Adult Vasculitis

**Figure 4:**
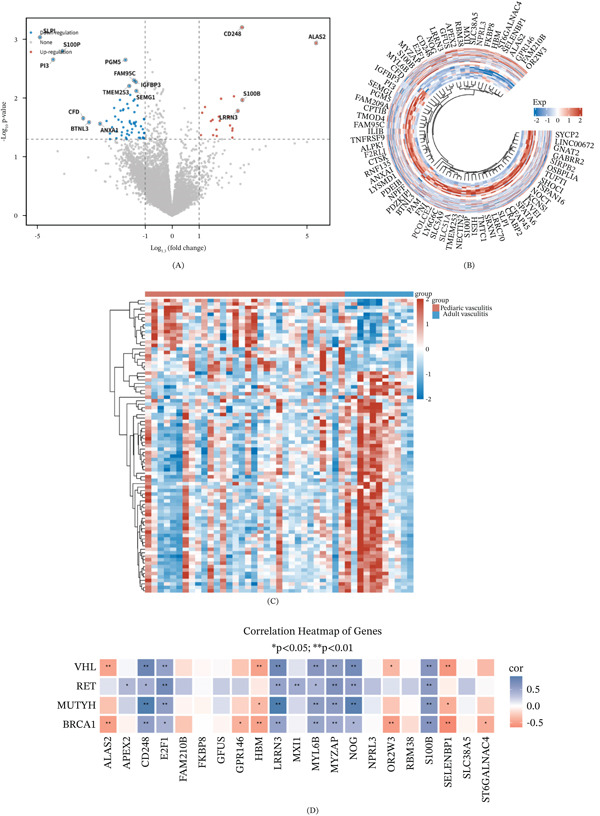
Differential transcriptomic landscape between pediatric and adult vasculitis. (A) Volcano plot showing differentially expressed genes between pediatric and adult vasculitis. (B) Circular heatmap displaying the expression patterns of representative DEGs across samples. (C) Hierarchical clustering heatmap based on DEGs distinguishing pediatric from adult vasculitis samples. (D) Correlation heatmap of germline mutation genes and hypoxia‐associated genes.

To further determine whether pediatric vasculitis exhibits a unique molecular phenotype compared with adult vasculitis, we performed differential expression analysis between the two cohorts. Volcano plot analysis identified a total of 85 DEGs, including 21 upregulated genes and 64 downregulated genes in pediatric vasculitis relative to adult vasculitis (Figure 4A). These findings indicate substantial age‐related transcriptional divergence in vasculitic disease biology. SLPI, PI3, and S100P showed prominent increases in pediatric vasculitis, suggesting enhanced epithelial defense, inflammatory modulation, neutrophil‐associated responses, or tissue repair–related programs. In contrast, CD248, S100B, and LRRN3 were among the genes downregulated in pediatric vasculitis and therefore relatively enriched in adult vasculitis (Figure 4A). To visualize overall expression patterns, circular heatmap and hierarchical clustering analyses were conducted. These results demonstrated clear separation between pediatric and adult vasculitis samples based on the identified DEGs, further supporting the existence of age‐dependent molecular heterogeneity (Figure 4B,C). Moreover, given the important contribution of germline mutations to disease susceptibility and inflammatory phenotypes, we further examined the correlations between highly expressed DEGs and core germline mutation–related genes. The correlation heatmap revealed a coordinated association pattern between several transcriptomic signatures and germline mutation genes. VHL and BRCA1 showed negative correlations with ALAS2, HBA1, OR2V23, STILENBP1, and ST6GALNAC4, whereas they were positively correlated with CD248, E2F1, LRRN3, MYL6B, MYZAP, NOG, and S100B. RET and MUTYH displayed broadly similar correlation profiles (Figure 4D).

### 3.5. Functional Enrichment Analysis Reveals Divergent Biological Programs Between Pediatric and Adult Vasculitis

**Figure 5:**
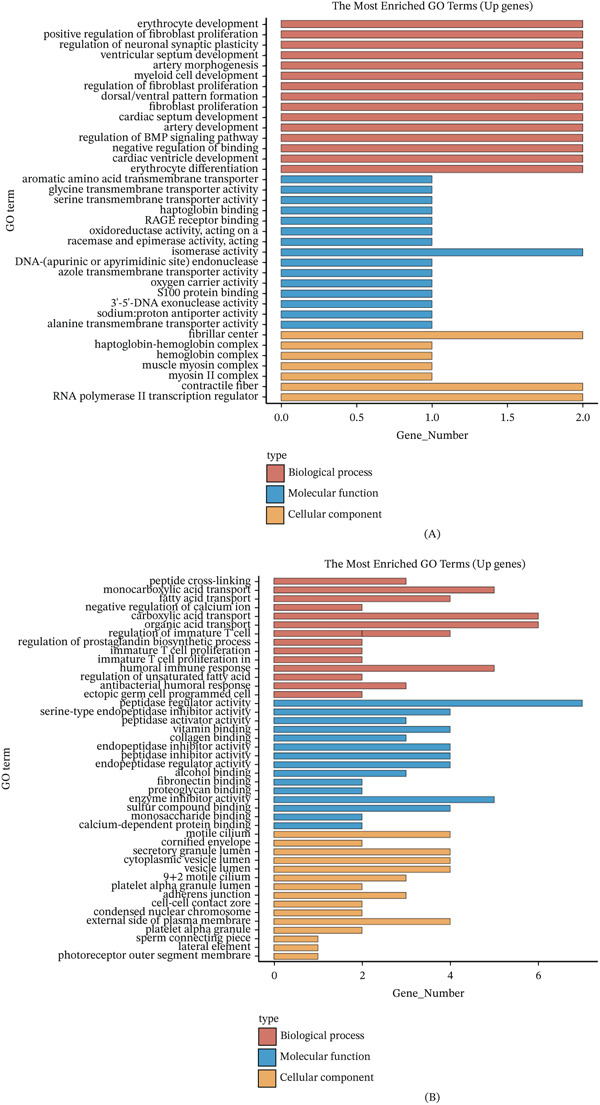
GO enrichment analysis of genes differentially expressed between pediatric and adult vasculitis. (A) Top enriched GO terms for genes upregulated in pediatric vasculitis compared with adult vasculitis. (B) Top enriched GO terms for genes downregulated in pediatric vasculitis (relatively enriched in adult vasculitis).

**Figure 6:**
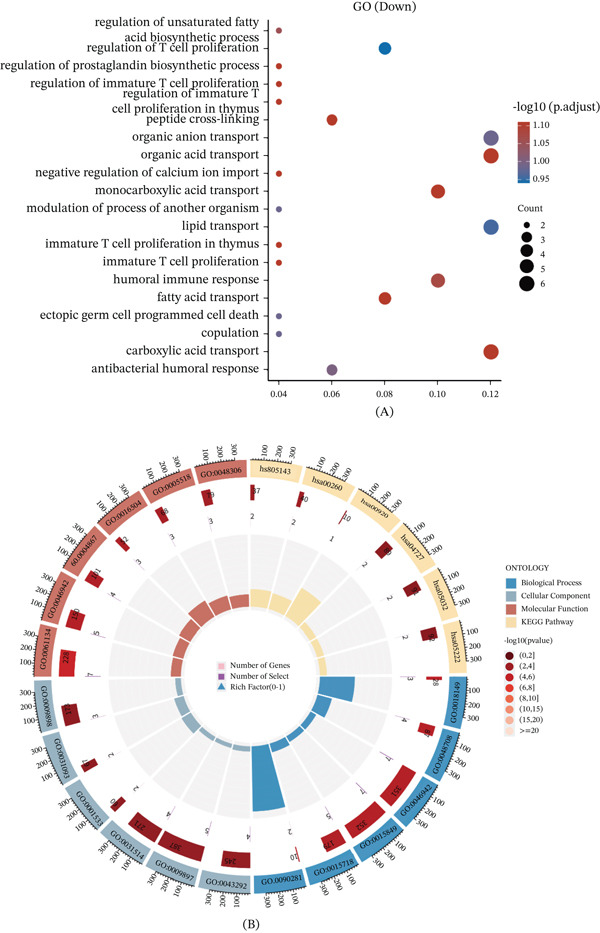
Advanced functional enrichment visualization of genes enriched in adult vasculitis. (A) Bubble plot showing representative enriched GO biological processes in adult vasculitis. (B) Circular enrichment plot integrating GO categories and pathway annotations.

To further explore the biological significance of age‐associated transcriptional differences, we performed GO enrichment analysis on the DEGs between pediatric and adult vasculitis. Functional annotation demonstrated that genes upregulated in pediatric vasculitis were predominantly enriched in pathways related to fibroblast proliferation, regulation of fibroblast proliferation, artery development, cardiac septum development, ventricular septum development, erythrocyte differentiation, and BMP signaling regulation (Figure 5A). These findings suggest that pediatric vasculitis may exhibit stronger vascular remodeling, stromal activation, and tissue developmental responses. In contrast, genes enriched in adult vasculitis were mainly associated with regulation of immature T cell proliferation, immature T cell proliferation in thymus, humoral immune response, regulation of prostaglandin biosynthetic process, and negative regulation of calcium ion import (Figures 5B and 6A). These results imply that adaptive immune remodeling and inflammatory mediator regulation may play more prominent roles in adult vasculitis. Additionally, molecular function and pathway analyses revealed significant enrichment of fatty acid transport, lipid transport, monocarboxylic acid transport, and related metabolic processes in adult vasculitis (Figure 6B), indicating that metabolic reprogramming may represent a distinguishing biological characteristic of adult disease. Collectively, these enrichment analyses demonstrate that pediatric vasculitis is more strongly associated with stromal proliferation and vascular developmental programs, whereas adult vasculitis is characterized by immune regulation and lipid metabolic signaling.

## 4. Discussion

Vasculitis is increasingly recognized as a biologically heterogeneous syndrome rather than a single inflammatory entity [48, 49]. In the present study, transcriptomic profiling based on hypoxia‐related genes divided pediatric vasculitis into two reproducible molecular subtypes, indicating that oxygen‐stress programs may represent an important axis of disease stratification. This finding is biologically plausible because inflamed vascular tissues often develop regional hypoxia as a result of endothelial swelling, luminal narrowing, leukocyte infiltration, and microthrombotic obstruction [50, 51]. Under these conditions, HIF‐1*α* and HIF‐2*α* can promote glycolytic reprogramming, VEGF‐mediated vascular responses, adhesion molecule expression, and inflammatory cytokine production [52–54]. Through interaction with NF‐*κ*B, JAK, PI3K, MAPK, and TLR signaling, hypoxia may further sustain endothelial activation, neutrophil recruitment, macrophage polarization, and vascular remodeling [55, 56]. Our findings suggest that hypoxia‐associated transcriptional programs are not merely downstream consequences of inflammation, but may define biologically distinct pediatric vasculitis endotypes.

A major feature of the high_hypoxia pediatric vasculitis group was the coordinated upregulation of immune checkpoint molecules, including PDCD1, LAG3, TIGIT, HAVCR2 (TIM‐3), and SIGLEC15. Although these molecules are commonly studied in cancer and chronic infection, they also participate in the regulation of inflammatory vascular injury by modulating T cell activation, myeloid‐cell responses, cytokine tone, and tissue damage. In a hypoxic inflammatory microenvironment, persistent antigenic stimulation, IL‐6, TNF‐*α*, Type I interferon signaling, and NF‐*κ*B activation may promote checkpoint expression in T cells and myeloid populations [57]. Thus, the checkpoint‐high subtype may represent a state of compensatory immunosuppression superimposed on sustained inflammation. Such a model may help explain why some vasculitis patients exhibit refractory disease despite systemic immunosuppression: inflammatory burden and immune paralysis may coexist [58]. This raises the possibility that checkpoint patterns could serve as biomarkers of immune state rather than immediate therapeutic targets.

We also observed enrichment of ferroptosis‐associated genes (EMC2 and ATP5MC3) together with broad activation of m6A regulators (FTO, METTL14, WTAP, and RBM15) in high_hypoxia pediatric vasculitis group. Ferroptosis, an iron‐dependent lipid peroxidation–driven cell death program, has emerged as a potent contributor to endothelial damage and sterile inflammation [33, 59]. Oxidative stress within inflamed vessels may sensitize endothelial cells, smooth muscle cells, and infiltrating leukocytes to ferroptotic injury, thereby perpetuating vascular necrosis and cytokine release [60]. Meanwhile, m6A methylation dynamically regulates mRNA stability and translation of inflammatory transcripts, metabolic enzymes, and stress‐response genes [61–63]. Importantly, recent studies in other diseases indicate crosstalk between hypoxia, m6A modification, and ferroptosis through HIF‐1*α*–METTL3/14 or FTO‐dependent pathways [39, 64, 65]. The high_hypoxia group may reflect an integrated hypoxia–immune checkpoint–ferroptosis–m6A regulatory state. Hypoxia may stabilize HIF‐1*α* and cooperate with NF‐*κ*B and STAT3 signaling to enhance inflammatory cytokines and checkpoint molecules, including PDCD1, LAG3, HAVCR2, TIGIT, and SIGLEC15. Concurrent oxidative stress and mitochondrial dysfunction may increase ferroptosis susceptibility through lipid peroxidation and impaired SLC7A11–GSH–GPX4 antioxidant defense. The upregulation of m6A regulators further suggests that posttranscriptional RNA modification may regulate HIF signaling, cytokine production, immune exhaustion, and ferroptosis‐related transcripts, thereby contributing to the coordinated molecular phenotype observed in high_hypoxia pediatric vasculitis group. Therefore, our data suggest that similar regulatory coupling may operate in vasculitis and warrants mechanistic validation.

Finally, comparison between pediatric and adult vasculitis revealed age‐dependent biology. Pediatric disease was enriched for fibroblast proliferation and vascular developmental programs, whereas adult disease showed stronger T cell regulatory and fatty acid transport signatures. These distinctions may reflect developmental immunity, vascular plasticity, and immunometabolic aging [66–68]. Children may mount more remodeling‐oriented responses, whereas adults exhibit chronic adaptive immune activation and lipid‐driven inflammation [67, 69, 70]. In addition to hypoxia‐associated subtype heterogeneity and age‐related transcriptional divergence, our analysis also identified coordinated correlations between highly expressed DEGs and germline mutation–related genes, including VHL, BRCA1, RET, and MUTYH. These findings suggest that germline susceptibility may represent an additional biological layer contributing to vasculitis heterogeneity.

Several limitations should be acknowledged. First, this study is based primarily on transcriptomic datasets and bioinformatic analyses; therefore, the observed relationships among hypoxia‐associated molecular subtypes, immune checkpoint genes, ferroptosis‐related genes, and m6A regulators should be interpreted as correlative rather than causal. Although the high_hypoxia pediatric vasculitis subtype showed coordinated upregulation of immune checkpoint molecules, ferroptosis‐associated genes, and m6A regulators, these findings do not prove that hypoxia directly drives immune exhaustion, ferroptotic vulnerability, or epitranscriptomic remodeling in pediatric vasculitis. Moreover, GO analysis is dependent on existing database annotations, which may be incomplete, biased toward well‐studied genes, or insufficiently specific for pediatric vasculitis biology. Lastly, further in vitro and in vivo studies integrating single‐cell, spatial transcriptomic, and large independent pediatric vasculitis cohorts are needed to further validate whether hypoxia‐checkpoint‐ferroptosis‐m6A networks can predict relapse, organ injury, or therapeutic responsiveness. Although our study is limited by retrospective public datasets and lack of prospective validation, it provides a framework for precision subclassification for routine diagnosis, prognosis assessment, and targeted therapy selection in pediatric vasculitis. Future studies integrating single‐cell, spatial transcriptomic, and longitudinal clinical data may determine whether hypoxia‐checkpoint‐ferroptosis‐m6A networks can predict relapse, organ injury, and therapeutic responsiveness.

## 5. Conclusion

In summary, this study identifies marked hypoxia‐associated molecular heterogeneity in pediatric vasculitis and stratifies patients into two biologically distinct subtypes with divergent immune checkpoint, ferroptosis‐related, and m6A epitranscriptomic profiles. The checkpoint‐high pediatric vasculitis subgroup may represent an integrated hypoxic inflammatory state characterized by HIF‐related signaling, cytokine amplification, immune exhaustion‐like regulation, oxidative stress, ferroptosis susceptibility, and dynamic posttranscriptional control. These findings suggest that hypoxia is not simply a secondary consequence of vascular inflammation, but may actively shape immunometabolic and epitranscriptomic remodeling in pediatric vasculitis.

## Author Contributions

**Bin Liu**: formal analysis, data curation, investigation, software, methodology, visualization, conceptualization, supervision, validation, writing–original draft, writing—review and editing.

## Funding

No funding was received for this manuscript.

## Consent

Each author gave the consent for publication.

## Conflicts of Interest

The author declares no conflicts of interest.

## Data Availability

The data in this study are available from the corresponding author upon reasonable request.
